# Identifications of Captive and Wild Tilapia Species Existing in Hawaii by Mitochondrial DNA Control Region Sequence

**DOI:** 10.1371/journal.pone.0051731

**Published:** 2012-12-12

**Authors:** Liang Wu, Jinzeng Yang

**Affiliations:** 1 Department of Human Nutrition, Food and Animal Sciences, University of Hawaii at Manoa, Honolulu, Hawaii, United States of America; 2 College of Animal Science & Technology, Huazhong Agricultural University, Wuhan, China; Auburn University, United States of America

## Abstract

**Background:**

The tilapia family of the Cichlidae includes many fish species, which live in freshwater and saltwater environments. Several species, such as *O. niloticus*, *O. aureus,* and *O. mossambicus*, are excellent for aquaculture because these fish are easily reproduced and readily adapt to diverse environments. Historically, tilapia species, including *O. mossambicus, S. melanotheron, and O. aureus*, were introduced to Hawaii many decades ago, and the state of Hawaii uses the import permit policy to prevent *O. niloticus* from coming into the islands. However, hybrids produced from *O. niloticus* may already be present in the freshwater and marine environments of the islands. The purpose of this study was to identify tilapia species that exist in Hawaii using mitochondrial DNA analysis.

**Methodology/Principal Findings:**

In this study, we analyzed 382 samples collected from 13 farm (captive) and wild tilapia populations in Oahu and the Hawaii Islands. Comparison of intraspecies variation between the mitochondrial DNA control region (mtDNA CR) and cytochrome c oxidase I (COI) gene from five populations indicated that mtDNA CR had higher nucleotide diversity than COI. A phylogenetic tree of all sampled tilapia was generated using mtDNA CR sequences. The neighbor-joining tree analysis identified seven distinctive tilapia species: *O. aureus, O. mossambicus, O. niloticus, S. melanotheron, O. urolepies, T. redalli,* and a hybrid of *O. massambicus* and *O. niloticus*. Of all the populations examined, 10 populations consisting of *O. aureus, O. mossambicus, O. urolepis,* and *O. niloticus* from the farmed sites were relatively pure, whereas three wild populations showed some degree of introgression and hybridization.

**Conclusions/Significance:**

This DNA-based tilapia species identification is the first report that confirmed tilapia species identities in the wild and captive populations in Hawaii. The DNA sequence comparisons of mtDNA CR appear to be a valid method for tilapia species identification. The suspected tilapia hybrids that consist of *O. niloticus* are present in captive and wild populations in Hawaii.

## Introduction

With easy breeding and high survival rates, the tilapia family of the Cichlidae has been an excellent species for aquaculture. Tilapia is one of the most widely farmed fish in the world with primary production from developing countries in Asia. In these countries, tilapia aquaculture not only provides dietary sources of protein and minerals for millions of impoverished families but is also an important means for economic and social empowerment. More than 2000 species of tilapia exist in both aquaculture and wild populations. Some of the species, such as *O. niloticus*, *O. aureus,* and *O. mossambicus*, are excellent for farming because these fish are easily bred and readily adapt to salty and alkaline environments [1], [2]. For example, Mosambique tilapia (*O. mossambicus*) and its hybrids can tolerate high salinity and are increasingly used in co-culture with marine shrimp [3], [4]. Strains of tilapia, such as *O. aureus*, *O. mossambicus,* and *S. melanotheron,* were introduced to Hawaii several decades ago. In the 1950 s and 1960 s, *O. mossambicus*, *S. melanopleura,* and *T. melanopleuron* were imported to the Hawaiian Islands to control vegetation and for use as baitfish for the tuna fishery. Taiwan and Florida red hybrid tilapias were also imported in the early 1980 s [5], [6]. Backcrossing and hybridization of the red tilapia with other stocks has been tested. The imported tilapia are thought to have entered the freshwater and marine environments along the recreational beaches, as these tilapia have been observed in local rivers, reservoirs, and brackish water in Hawaii.

A lack of genetically suitable tilapia broodstocks has been a limiting factor for tilapia aquaculture in Hawaii. Importation of genetically-selected tilapia strains to Hawaii has been challenged by environmental concerns and field-testing requirements. Currently, the State of Hawaii requires a permit to import and raise *O. niloticus* and the hybrids. Over the years, aquarists have released a remarkable number of tilapias into Hawaii's streams and reservoirs. These tilapias that exist in the wild and on farms can be used as genetic resources for developing high-growth tilapia without importing new strains. Moreover, reproduction by random breeding in aquaculture practices may reduce the genetic diversity in domesticated strains due to the inbreeding effects of small broodstock population size [7], [8]. Local tilapia populations used for aquaculture are actually suffering from inbreeding depression. Therefore, understanding the tilapia species structure in wild populations and farm stocks is important. Therefore, we sought to identify the tilapia species that exist in the wild and captive populations in Hawaii.

The traditional distinction of species within tilapia family depends on the differences in appearances of characteristics such as body size, shape, color, number of anal spines, shape of fins, and color of the head. However, introduction of alien species and the hybridization between these species have made identification of tilapia species by morphological distinctions more complicated [2], [3]. Compared with morphological observation and description, a DNA-based approach is accurate and practical. The sequence of a single mitochondrial protein-coding gene, namely cytochrome c oxidase subunit I (COI), is used as a platform for DNA barcoding of all living species [9]–[11]. Several DNA marker systems have also been reported for tilapia species identification, including microsatellite markers, 45 s and 5 s rDNA, and the mitochondrial DNA control region (mtDNA CR) [12]–[18]. The mtDNA CR, which is located between the tRNA-glu gene and the tRNA-phe gene, is the most variable part of the mtDNA and evolves three to five times more rapidly than the rest of the mitochondrial genome. This segment of mitochondrial DNA sequence has been used for classification and describing phylogenic relationships of 42 tilapiine species originated from Africa [2]. In this study, we report the identification of tilapia species existing in both captive and wild populations in Hawaii by using mtDNA CR sequence data which is then compared with previously published data.

## Results

### Tilapia Species Identification by mtDNA CR and COI Sequences

**Figure 1:**
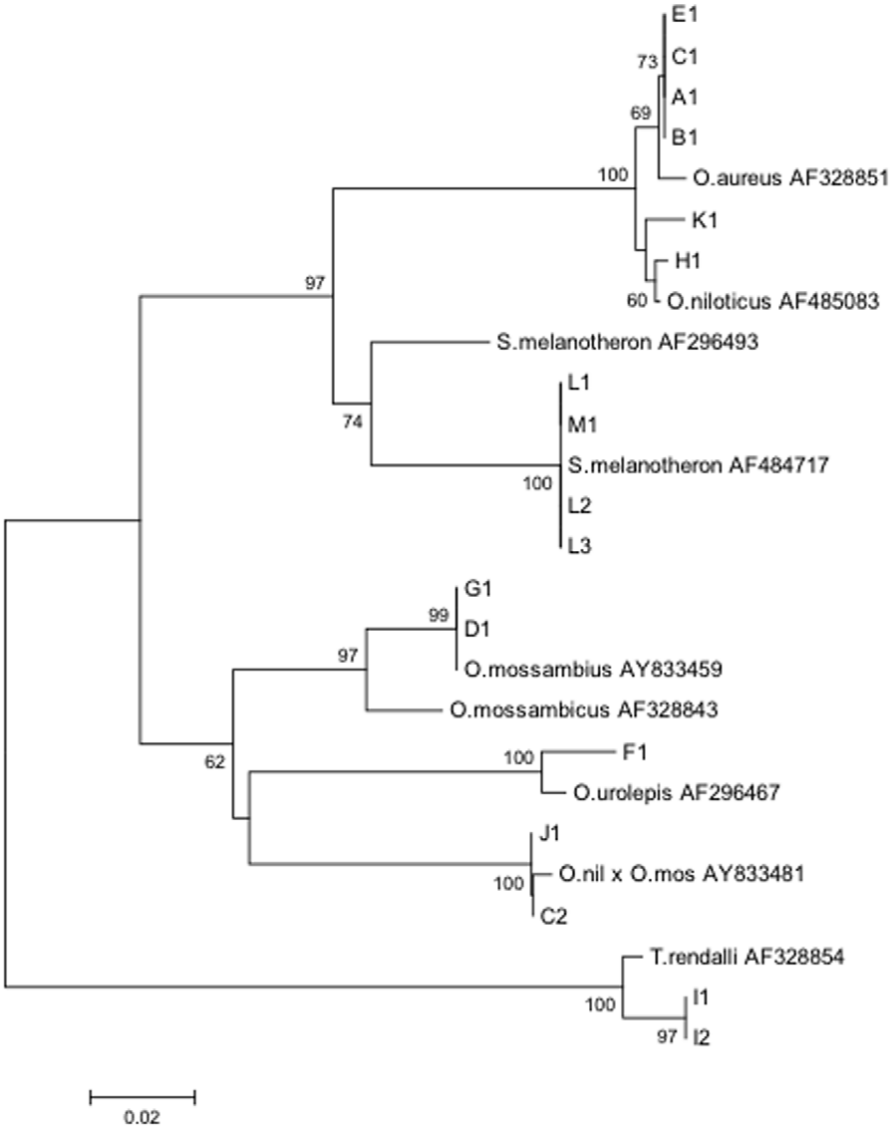
Phylogenetic tree constructed with tilapia mtDNA CR sequences using the Kimura two-parameter distance model. Bootstrap values greater than 50 are shown. A1, B1, C1, C2, D1, E1, F1, G1, H1, I1, I2, J1, K1, L1, L2, and L3 are the representative mtDNA CR sequence from different fish populations. *O. niloticu* x *O. mossambic* (accession #AY833481), *O. mossambic* (accession #AY833459), *O. urolepis* (accession #AF296467), *O. aureus* (accession #AF328851), *O. niloticus* (accession #AF485083), *S. melanotheron* (accession #AF484717), and *T. rendalli* (accession #AF328854) are reference tilapia species, and the accession numbers were obtained from the NCBI database.

**Figure 2:**
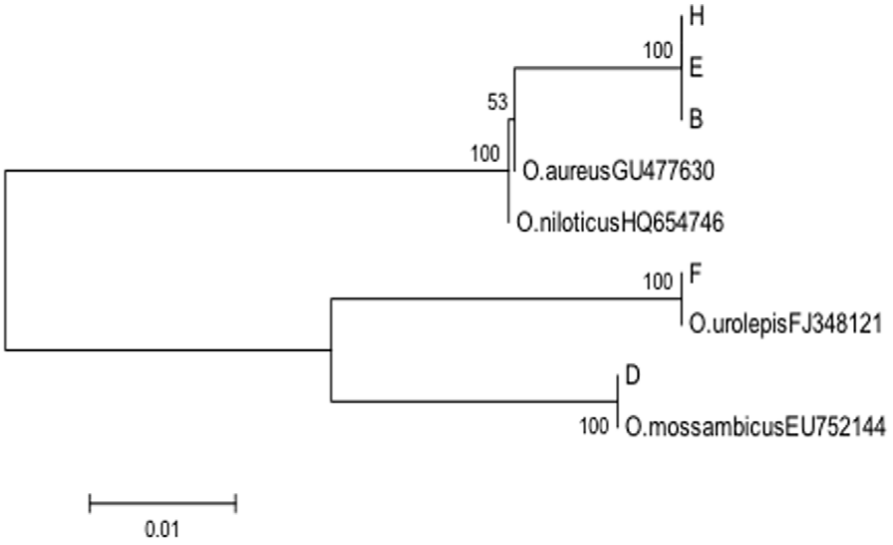
Phylogenetic tree constructed with tilapia mtDNA COI gene sequences using the Kimura two-parameter distance model. Bootstrap values greater than 50 are shown. B, D, E, F, and H are the representative COI gene sequences from different fish population. *O. aureus* (accession #GU477630), *O. nioticus* (accession #HQ654746), *O. urolepis* (accession #FJ348121), and *O. mossambicus* (accession #EU752144) are reference tilapia sequences, and their accession numbers were obtained from the NCBI database.

**Figure 3:**
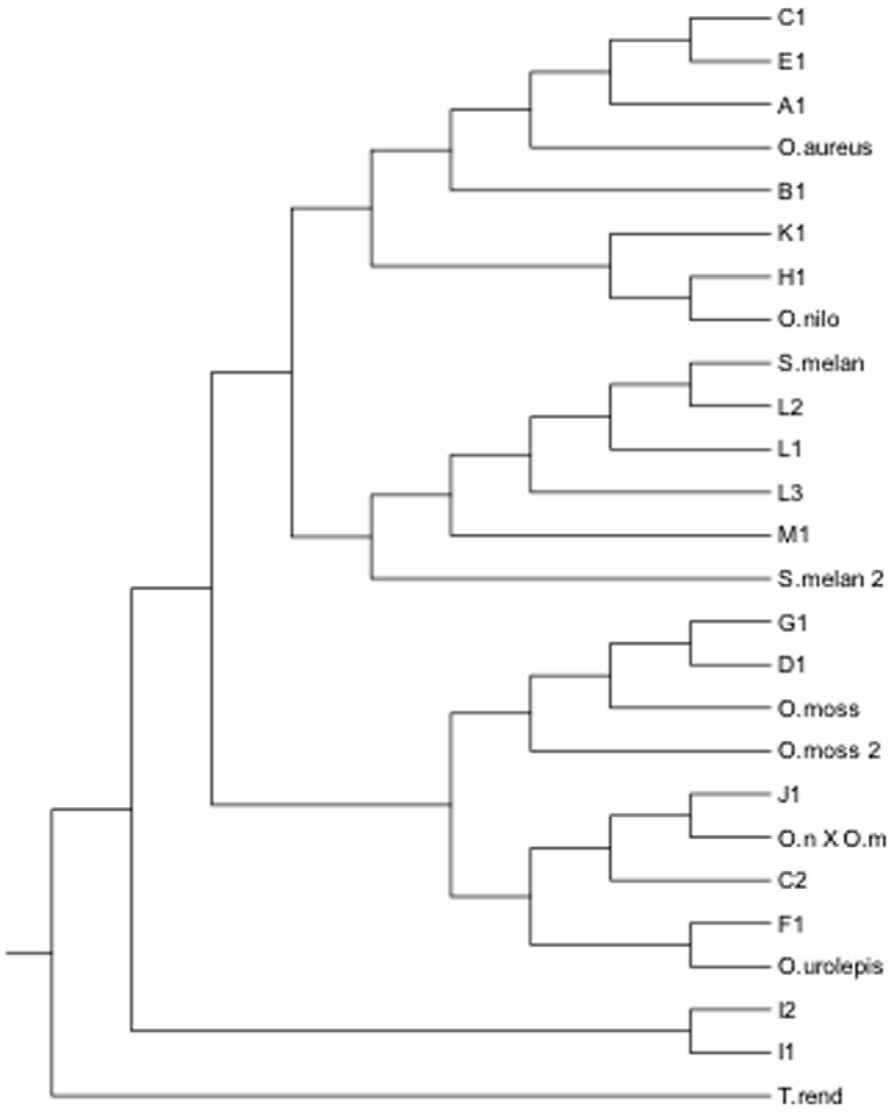
Phylogenetic tree constructed with tilapia mtDNA CR sequences by PHYLIP program. A1, B1, C1, C2, D1, E1, F1, G1, H1, I1, I2, J1, K1, L1, L2, and L3 are the representative mtDNA CR sequence from different fish populations. *O. niloticu* x *O. mossambic* (O.n x O.m, accession #AY833481), *O. mossambic* (O.moss, accession #AY833459 and O.moss 2, accession # AF328843), *O. urolepis* (accession #AF296467), *O. aureus* (accession #AF328851), *O. niloticus* (O. nilo, accession #AF485083), *S. melanotheron* (S. melan accession #AF484717 and S. melan 2 accession # AF296493), and *T. rendalli* (T. rend, accession #AF328854) are reference tilapia species, and the accession numbers were obtained from the NCBI database.

The genetic distances of five populations were calculated to compare the identification of tilapia species using mtDNA COI and mtDNA CR sequences. The mtDNA CR exhibited a higher genetic distance between the species than COI did. The genetic distances between population H and E, population B and E, and population B and H were found to be 0.000 using COI DNA sequence, while the genetic distances between population H and E, population B and E, and population B and H are 0.0125, 0.0025, and 0.0150, respectively (Table 1 and Table 2). The phylogenetic tree generated from mtDNA CR sequences successfully differentiated population H (*O. niloticus*) from population E and B, both of which are species of *O. aureus* (Figs 1 and 2) while the phylogenetic tree based on mtDNA COI data failed to differentiate the species *O. aureus* and *O. niloticus*. Therefore, mtDNA CR shows higher nucleotide diversity than mtDNA COI in the five populations currently being studied. Thus, we chose individual fish mtDNA CR as a valid DNA marker for tilapia species identification. In addition, mtDNA COI was used as a genetic marker to test the different samples for a cross-reference check and validation of the classified tilapia species in this study. Except the clustering of the species *O. niloticus* and *O. aureus*, all other tilapia species clusterings based on the COI sequence data are consistent with the results from the mtDNA CR sequence data. Moreover, the phylogenetic tree (Fig. 3) constructed by program PHYLIP with maximum likelihood method also shows a similar result to the neighbor-joining tree (Fig. 1) was constructed with the Kimura Two-parameter distance model by MEGA Version 4.

**Table 1:** The genetic distance between different populations based on mtDNA CR sequence.*.

Population	D	F	E	B
F	0.0872			
E	0.1296	0.1442		
B	0.1328	0.1475	0.0025	
H	0.1294	0.1440	0.0125	0.0150

* MtDNA CR genetic distances among different population were obtained by MEGA Version Data are presented by intraspecific or interspecific congeneric K2P-distances.

**Table 2:** The genetic distance between different populations based on mt DNA COI sequence.*.

Population	E	F	D	H
F	0.0775			
D	0.0737	0.0423		
H	0.0000	0.0775	0.0737	
B	0.0000	0.0775	0.0737	0.0000

* MtDNA CR genetic distances among different population were obtained by MEGA Version4. Data are presented by intraspecific or interspecific congeneric K2P-distances.

### Tilapia Species Identification

**Figure 4:**
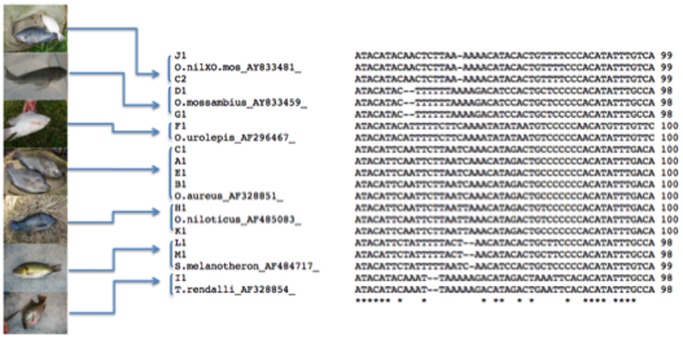
Alignments of the variable sites of mtDNA CR sequences from the representative fish populations. Dashes indicate indels introduced for optimal alignment, and the asterisks at the bottom of the figure indicate the consensus nucleotide. A1, B1, C1, C2, D1, E1, F1, G1, H1, I1, I2, J1, K1 and L1 are the representative mtDNA CR sequence types from different fish populations.

**Figure 5:**
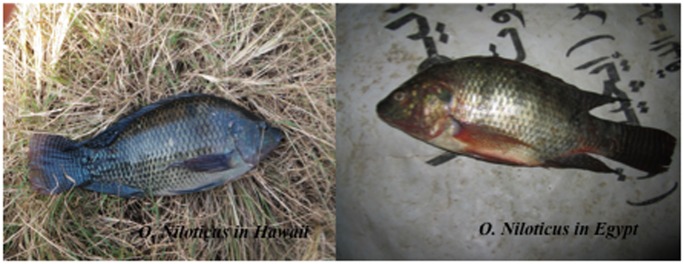
Comparisons between *O. niloticus* present in Hawaii and *O. niloticus* in Egypt.

A total of 420 tilapia fin samples were collected from 13 populations from local farm and wild population sites in Hawaii. Genomic DNA was extracted from the fin clip samples of 390 fish and used for PCR amplification of mtDNA CR sequence. 382 fish samples were sequenced (Table 3) and compared with the reported tilapia mtDNA CR sequences (Fig. 4). All the fish samples were classified to the related tilapia species identities. Of the 13 populations, seven different tilapia species and one hybrid were identified, including *O. aureus, O. mossambius, O. urolepis, O. niloticus, S. melanotheron, T. rendali,* and based on the similarity of mtDNA CR sequence, 1 population was recognized as hybrid of *O. niloticus×O. mossambicus* which was reported by D’ Amato [19]. The results from this study confirm that *O. niloticus* (Fig. 5) and its hybrids exist in the wild and captive sites in Hawaii. A phylogenetic tree that was constructed on the basis of the mtDNA CR sequences was rooted using the K2P/NJ model taking in to account transitional and transversional substitution rates at the midpoint. All of the sequences were successfully differentiated to tilapia species by the phylogenetic tree, and most of these had a bootstrap value above 90%. According to the phylogenetic tree, the sequences from the 13 populations were separated into seven tilapia species. The genetic distances between the groups and within the groups were calculated by MEGA. The nucleotide diversity of individuals between groups ranged from 0 to 0.3874.

**Table 3:** Identifications of Captive and Wild Tilapia by mtDNA CR Sequence.

Location	Code of the Population	Number of FishSequenced	Species Identities
Aquaculture Site	A	30	*O. aureus*
Aquaculture Site	B	28	*O. aureus*
Aquaculture Site	C	29	*O. niloticus×O. mossambicus and O.aureus*
Aquaculture Site	D	29	*O. mossambius*
Aquaculture Site	E	30	*O. aureus*
Aquaculture Site	F	30	*O.urolepis*
Aquaculture Site	G	29	*O.mossambicus*
Aquaculture Site	H	30	*O. niloticus*
Aquaculture Site	J	28	*O. urolepis*
A local stream in Hilo	K	30	*O. niloticus*
Nuuanu Reservoir	L	30	*S. melanotheron*
Nuuanu Reservoir No.2	I	30	*T.rendalli*
Wahiawa Lake	M	29	*S. melanotheron*

Of the seven distinct species groups, *O. aurues* and *O. mossambicus* were the most popular fish in the studied populations. *O. aurues* included 88 fish samples from three populations. *O. mossambicus* included 58 fish samples from two populations. According to the mtDNA nucleotide diversity value, the samples collected from domesticated populations demonstrated a very low genetic diversity (most of them are zero) and the samples from wild population had higher nucleotide diversity. These data suggest that the farm populations except population C have low genetic variabilites within the population, whereas the wild populations demonstrated various degrees of introgression and hybridization. The interspecies genetic distances and average intraspecies genetic distances were calculated with MEGA 4. The mean distance among the total sample is 0.103. The distance within species ranged from 0 to 0.072, while the distance between species ranged from 0.012 to 0.210. Significant variation of the genetic distance between the interspecies and intraspecies comparisons was noted. Seventeen different haplotypes were detected from the analyzed mtDNA CR sequences. Sample number, haplotype numbers, average number of nucleotide differences, and nucleotide diversity are shown in Table 4. Compared with the samples from aquaculture sites, the wild populations had a high haplotype number.

**Table 4:** Genetic variability in captive and wild populations.*.

Population	N	Pi	H	K
A	30	0.000	1	0.000
B	28	0.000	1	0.000
C	29	0.38748	2	149.179
D	29	0.000	1	0.000
E	30	0.000	1	0.000
F	30	0.000	1	0.000
G	29	0.000	1	0.000
H	30	0.000	1	0.000
J	28	0.000	1	0.000
K	30	0.000	1	0.000
L	30	0.24400	3	121.532
I	30	0.21342	2	98.821
M	29	0.000	1	0.000

* N: the number of sequences; Pi: nucleotide diversity within the population; H: number of different sequences types; K: average number of nucleotide differences within the population.

## Discussion

Several molecular-based approaches for the identification of tilapia species have been described, including microsatellite analysis, DNA barcoding, analysis of the nuclear fragment of rDNA, the first internal transcribed spacer, and mitochondrial DNA restriction fragments [20]–[23]. Compared with classical morphological methods, a molecular approach to species classification is more accurate and practical. Selection of quality and reliable DNA markers is becoming important for species identification. Based on the report from previous studies, COI and mtDNA CR were employed as candidate DNA markers. We used fish samples from 5 populations to compare both DNA markers. According to the result we found mtDNA CR had a higher degree of DNA variability than mt DNA COI. The mtDNA COI sequence is widely used for fish identification as a standard DNA barcoding method [24]–[30]. However, recent research indicated that the COI failed to distinguish closely-related species due to their lower genetic variability [12]. Similarly, our results also demonstrated that COI failed to distinguish *O. aureus* and *O. niloticus* in the sampled tilapia populations. However the species identification based on mtDNA CR was unambiguous. Therefore, mtDNA CR was used for all the fish samples collected from the wild and captive populations. Our data suggest that mtDNA CR sequence provides sufficient genetic variability for tilapia species identifications for our sampled populations. Based on the mtDNA CR sequences, we identified seven distinctive tilapia species, including *O. aureus, O. mossambicus, O.niloticus, S. melanotheron, O. urolepies, T. redalli,* and a hybrid of *O. massambicus* and *O. niloticus*. The suspected *O. niloticus* and hybrids are present in captive and wild populations in Hawaii.

*O. mossambicus* was probably the first tilapia species to be widely distributed in Hawaii. As a result, most *O. mossambicus* stocks were unmanaged, and imported stocks of this species have escaped to the wild and been established in all the major islands of Hawaii. This species spawns easily in seawater as well as freshwater. Early experiences in aquaculture of this species were unappreciated as a food fish due to its large head, slow growth rates, and small body size at harvest [31], although *O. mossambicus* still has the potential to contribute to the breeding of tilapia. For example, Ch’ang described a remarkable improvement of the growth rate and weight gain at 5 months of *O. mossambicus* under aquaculture conditions [32]. In the 1990’s, *O. aureus* was imported to Hawaii from the US Mainland for research purposes, and a hybrid of *O. aureus* and *O. niloticus* (Rocky Mountain White) was imported in 1995 by a commercial farmer [33]. Fish from Population C were identified to contain the DNA sequences of both *O. mossambicus x O. niloticus* and *O. aureus*, and this population may be related to the imported hybrids, which had been imported into Hawaii from Taiwan in 1980 [3]. Based on the genetic diversity and phylogenetic tree analysis, we found that Population K showed genetic characteristics of *O. niloticus*. Clearly, this study confirm that *O. niloticus* and hybrids are present in the wild and captive sites in Hawaii.

**Figure 6:**
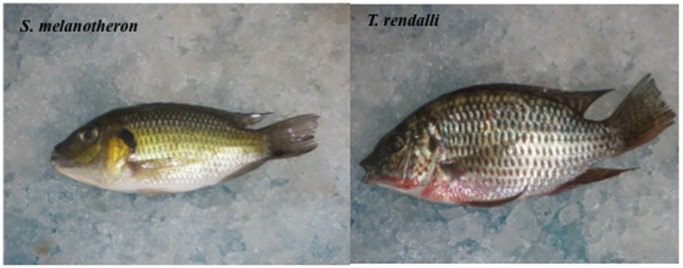
Tilapia species *S. melanotheron* and *T. rendalli*.

The results of phylogenetic tree analysis demonstrated that the populations collected from the wild reservoirs included two different species, *S. melanotheron* and *T. rendalli* (Fig. 6). Measures of the population nucleotide diversity revealed six haplotypes in the *S. melanotheron* population and two haplotypes in the *T. rendalli* population. According to the records from the state, *S. melanotheron*, which is known as blackchin tilapia, was introduced into Hawaii in 1962 by the US federal government fishery services. The introduction of this species occurred in order to test this species as a baitfish for tuna [3]. Blackchin tilapia has the ability to survive in pure seawater. Apparently, the fish escaped to the wild seawater environments, such as those in the coastal and lagoon waters, as well as to the local reservoirs in Hawaii.

Of the populations examined, the wild populations showed varying degrees of introgression and hybridization. In population K (*O. Niloticus*) and M (*S. Melanotheron*), nucleotide diversity within the population (Pi) and average number of nucleotide differences within the population (K) in Table 4 are zero. We don’t know why there is not any variability in population K and M. We collected the fish samples from Nuuanu Reservoir No. 1 and No.2 in Oahu Island. There is a river connect two reservoir so it is possible that there are gene flow between those tow populations. Population M was collected from Wahiawa lake which is located in North side of the Island. Considering both geographical distance and sequence variability suggest that this population is not introgressed. The other two wild populations *T. rendalli* and *S. Melanotheron have high nucleotide diversity and average number of nucleotide differences within population (* *Table 4* *). T. rendalli* was one of the earliest imported tilapia species, which was introduced to Hawaii from the Belgian Congo in Africa in 1957 [31]. According to records from the state marine biologists, *T. rendalli* were stocked in a local reservoir by the state in the late 1950 s for weed control. Initially, only 57 fish were imported, and the stockfish were bred in captivity and later stocked in reservoirs and canals [3]; however, DNA data analysis from the sampled fish from the wild reservoir indicated a high diversity of nucleotide variation compared with other species. This finding indicates that the population *T. reandalli* may be introgressed with other species. In many cases, genetic introgression was the result of intentional or negligent crossbreeding. This conclusion is based on a single mtDNA sequence analysis, and thus, further sampling and DNA analysis are certainly needed to verify this conclusion.

According to the mtDNA nucleotide diversity value, the samples collected from farm sites demonstrated very low genetic diversity. These results are likely a result of inbreeding and a limited number of founder stocks in the populations. Breeding methods inevitably influence the genetic variability of populations. Heterozygote deficiency appeared in black-chinned tilapia because of related adults mating [11]. Similar results have also been observed in the kin relationships of parents match experiences [17]. Minimizing the mating of closely related individuals has been suggested to be very helpful for maintaining the genetic diversity of a population [11]. Genetic bottlenecks exist widely in fish aquaculture operations with a small number of founder brood stocks at importation. For example, the initial *O. niloticus* stock brought to the Philippines originated from a small number of fish imported from Thailand in 1972. Thailand’s nationwide stock is, in turn, derived from about 200 fish from Japan, and these fish were derived from ancestors collected from open waters in Egypt in 1962 [33]. As no pedigree records for the import and transportation of these fish were available, we can assume that these practices would inevitably result in inbreeding and associated low reproduction and growth performance.

## Materials and Methods

### Ethics Statement

The relevant permission for the capture of tilapia at the different sites, including the aquaculture facilities, for the observational and field studies, along with tilapia sampling and use for this study, were approved by the Institutional Animal Care and Use Committee of University of Hawaii under the protocol No. 10-1039-2 and the Animal Welfare Assurance No. A3423-01. Captive tilapia aquaculture was conducted in accordance with the national and international guidelines for animal welfare. For fin clip sampling from captive and wild sites, fish were caught with a fishing net and the dorsal fin (1–2 cm) was taken by scissors within 1 minute. The sampled fish were released back into the water.

### Collections of Fish Sample

Tilapia fin clip samples were collected from thirteen populations (sites), including five wild populations on the island of Oahu and eight aquaculture facilities. Fin clip samples from 30–50 fish were collected from each site, and possible species identifications or pictures of the tilapia fish were taken at the time of sample collection. A total of 420 tilapia fin samples were collected and preserved in 100% ethanol in −20°C, and 390 fish samples were used for the DNA analysis.

### DNA Extraction and PCR Amplifications

Genomic DNA was isolated from caudal fin clips. Briefly, tissue samples were digested with 0.5 g/l proteinase K at 55°C overnight. The resulting solution was centrifuged; the supernatant was extracted by phenol-chloroform and precipitated in ethanol and dissolved in 1X TE buffer [15]. The quality and concentration of DNA were assessed by spectrophotometer and agarose gel electrophoresis, and DNA samples were stored at 4°C until use. PCR for mtDNA CR was carried out by using the primer set ORMT-F: 5′-CTAACTCCCAAAGCTAGGAATTCT-3′, ORMT-R: 5′-CTTATGCAAGCGTCGATGAAA-3′. To confirm and validate the mt CR method, we also developed PCR and DNA sequencing protocols of mtDNA COI (DNA barcoding with PCR primer set of VF2: GTAAAACGACGGCCAGTCAACCAACCACAAAGACATTGGCAC.

FishR: CAGGAAACAGCTATGACACTTCAGGGTGACCGAAGAATCAGAA [16]. The total PCR reaction volume for mtDNA CR was 25 µL and included the following components: 2.5 µL 10×PCR buffer, 0.5 µL of each primer (0.01 mM), 0.5 µL of dNTPs (10 mM), 0.2 µL of Taq DNA polymerase (5U/µL), and 1.0 µL of template DNA (50 ng). The PCR program consisted of a denaturation step for 3 min at 94°C, followed by 35 cycles of 94°C for 30 s, 54°C for 40 s and72°C for40 s; and a final extension step of 72°C for 10 min. Negative controls were used in all PCR reactions to make sure that no contamination occurred during the reaction system. All PCR products of 450 bp fragment were separated on 1.5% agarose gels. Images were photographed under UV light with an imaging system. The agarose gel contained DNA bands was cut off and the DNA bands were recovered with the DNA purification kit (Fermentas, Glen Burnie MD). After purification, amplified PCR products were sequenced. The PCR protocol for mtDNA COI is very similar to the mtDNA CR except the PCR primers and cycle conditions (94?C for 5 min, 35 cycles of 94?C for 30 s, 50?C for 40 s, and 72?C for 1 min, with a final extension at 72°C for 10 min). The amplified fragments with expected size of 600 bp was checked by agarose gel electrophoresis and sequenced.

### Sequence Data Analysis

After purification of PCR products, amplified DNA products from 382 fish samples were sequenced. All sequences were compared with the reported tilapia mtDNA CR sequences and the fish samples were classified to the related tilapia species identities. All the sequences were aligned by a clustalW2 program and visually checked for optimization. There were approximately 390 sites for mtDNA CR and 625 sites for COI, including alignment gaps. All of those sites were analyzed with seven recognized species of tilapia including the *O. aureus, S. melanothern, T. rendalli, O. mossambicus, O. urolepis* reported by Nagl et al (Genbank Accession number AF328851, AF296493, AF328854,AF328843, AF296467, AF328843)?2?, *S. melanothern, O.niloticus* reported by Falk (Genbank Accession number AF484717, AF485083)?34?, *O. mossambicus* and hybrid tilapia reported by D’ Amato (Genbank Accession number AY833459, AY833481)?19?. A neighbor-joining (NJ) tree was constructed with the Kimura Two-parameter distance model by MEGA Version 4 [35] and PHYLIP with maximum likelihood method for all sequences [36]. All transitions and transversions were calculated in the tree. The branching order was tested by 500 bootstrap. Genetic distances were quantified within and among species using the Kimura two-parameter (K2P) distance model by MEGE version 4.
